# Supplementary material for: Effects of Two Feeding Patterns on Growth Performance, Rumen Fermentation Parameters, and Bacterial Community Composition in Yak Calves
Source: Microorganisms. 2023 Feb 24;11(3):576. doi: 10.3390/microorganisms11030576 (PMC10058967; doi:10.3390/microorganisms11030576)
Supplement: Supplementary file 1 [file microorganisms-11-00576-s001.zip › microorganisms-2148392-supplementary.pdf]

microorganisms

Table S1. The level of phylum (top 10) and genus (top 20) in terms of relative abundance of microorganisms.

Taxonomic Level	Microbes	Group1		SEM	P-value
		CON	TRT		
Phylum	Bacteroidetes	36.78	58.33	10.78	0.001
	Firmicutes	55.47	37.01	9.23	0.003
	Actinobacteria	1.93	1.20	0.37	0.437
	Saccharibacteria	0.77	0.68	0.05	0.741
Genus	g__Prevotella_1	15.04	39.90	12.43	0.000
	g__unidentified	18.98	13.65	2.67	0.155
	g__Quinella	15.78	6.45	4.67	0.328
	g__Christensenellaceae_R-7_group	6.39	6.00	0.20	0.723
	g__Rikenellaceae_RC9_gut_group	5.23	4.17	0.53	0.413
	g__Succiniclasticum	3.72	2.73	0.50	0.398
	g__Ruminococcaceae_NK4A214_group	2.62	2.52	0.05	0.881
	g__Butyrivibrio_2	2.01	1.83	0.09	0.765
	g__Prevotellaceae_UCG-003	2.46	1.17	0.65	0.026
	g__Prevotellaceae_UCG-001	1.34	1.64	0.15	0.582
	g__Veillonellaceae_UCG-001	1.29	0.96	0.17	0.494
	g__Lachnospiraceae_NK3A20_group	0.97	1.30	0.17	0.389
	g__Olsenella	1.29	0.87	0.21	0.624
	g__Lachnospiraceae_XPB1014_group	0.69	1.44	0.38	0.005
	g__Pseudobutyrivibrio	1.29	0.74	0.28	0.170
	g__Ruminococcus_1	1.19	0.79	0.20	0.347
	g__Eubacterium_coprostanoli-genes_group	1.34	0.54	0.40	0.016
	g__Ruminococcaceae_UCG-014	0.85	0.96	0.06	0.537
	g__Saccharofermentans	0.84	0.77	0.04	0.760

[1]CON group: breastfeeding; TRT group: Milk Replacer feeding.
